# Design and development of a portable resistive sensor based on α‐MnO_2_/GQD nanocomposites for trace quantification of Pb(II) in water

**DOI:** 10.1049/nbt2.12042

**Published:** 2021-03-23

**Authors:** Amit K. Gupta, Mansi Khanna, Souradeep Roy, Pankaj, Shalini Nagabooshanam, Ranjit Kumar, Shikha Wadhwa, Ashish Mathur

**Affiliations:** ^1^ Amity Institute of Nanotechnology Amity University Uttar Pradesh India; ^2^ Department of Electronics and Communication Engineering Amity School of Engineering Amity University Uttar Pradesh India; ^3^ Department of Chemistry, School of Engineering University of Petroleum and Energy Studies Bidholi Campus Dehradun India; ^4^ Department of Physics, School of Engineering University of Petroleum and Energy Studies Bidholi Campus Dehradun India

## Abstract

The occurrence of heavy metal ions in food chain is appearing to be a major problem for mankind. The traces of heavy metals, especially Pb(II) ions present in water bodies remains undetected, untreated, and it remains in the food cycle causing serious health hazards for human and livestock. The consumption of Pb(II) ions may lead to serious medical complications including multiple organ failure which can be fatal. The conventional methods of heavy metal detection are costly, time‐consuming and require laboratory space. There is an immediate need to develop a cost‐effective and portable sensing system which can easily be used by the common man without any technical knowhow. A portable resistive device with miniaturized electronics is developed with microfluidic well and α‐MnO_2_/GQD nanocomposites as a sensing material for the sensitive detection of Pb(II). α‐MnO_2_/GQD nanocomposites which can be easily integrated with the miniaturized electronics for real‐time on‐field applications. The proposed sensor exhibited a tremendous potential to be integrated with conventional water purification appliances (household and commercial) to give an indication of safety index for the drinking water. The developed portable sensor required low sample volume (200 µL) and was assessed within the Pb(II) concentration range of 0.001 nM to 1 uM. The Limit of Detection (LoD) and sensitivity was calculated to be 0.81 nM and 1.05 kΩ/nM/mm^2^, and was validated with the commercial impedance analyser. The shelf‐life of the portable sensor was found to be ∼45 days.

## 1 INTRODUCTION

Heavy metal pollution is known to be one of the most serious pollution problems in nature because of the stability of metals at contaminated sites and high toxicity to the biosphere [1]. Among various heavy metal species, lead (Pb) has found numerous industrial applications, giving rise to the occurrence of toxic Pb^2+^ in the environment, especially affecting aquatic life and eventually migrating into the food chain [2]. Accumulation of Pb^2+^ in human bodies through the food chain and/or inadvertent exposure to the contaminating sources has been implicated in permanent neurological damage, inhibition of foetal development and malfunctioning of many organs including brain [2, 3, 4]. Some other complications arising due to Pb poisoning include severe hallucinations, vertigo, renal disorder, hypertension, paralysis and dyslexia and under chronic situations, often leading to death. Such life‐threatening consequences of Pb poisoning demands rapid monitoring of such species.

The conventional techniques commonly employed for the quantification of trace amounts of Pb in water include Inductive Coupled Plasma Mass Spectroscopy (ICP–MS) [5], Inductive Coupled Atomic Emission Spectroscopy (ICP–AES) [6] and Atomic Absorption Spectrometry (AAS) [7]. The incorporation of such sophisticated instrumentation is limited due to high equipment costs, requirement of skilled personnel, time‐consuming and tedious sample preparation protocols. Furthermore, owing to the bulkiness, cost factor and high power consumption, these instruments are not suitable for on‐site and real‐time monitoring of Pb concentrations. In contrast, electroanalytical strategies have provided relatively simple and low‐cost alternatives to realize rapid in situ detection of metal ions without the need for cost‐prohibitive specialized equipment, by employing nanostructured electrodes towards the development of real‐time Pb monitoring devices [8, 9, 10]. Such modules would then possess the potential for miniaturization, allowing user‐friendly access, less power consumption, reduced operation time, enhanced efficiency and ultimately reducing the overall device cost to a greater extent.

Recently, many inorganic materials have gained significant attention due to their low cost, compatibility and strong adsorption to heavy metal ions [11, 12]. A nanocomposite of reduced graphene oxide–bismuth nanoparticles (RGO/Bi) has been synthesized for sensitive detection of multiple heavy metals, in which the detection limits of 2.8, 0.55, 17 and 26 μg L^−1^ were obtained for Cd^2+,^ Pb^2+^, Zn^2+^ and Cu^2+^, respectively [13]. Toghill et al. (2009) prepared an antimony nanoparticle modified boron doped diamond electrode for simultaneous electrochemical determination of Pb^2+^ and Cd^2+^ over the range of 50–500 mg L^−1^ [14]. Quantum dots have also been explored for the detection of heavy metal ions, however, due to their toxicity, they have not been widely studied. Carbon nanotubes (CNTs) and Graphene require functionalization to introduce functional moieties on the surface. Nanostructured metal oxides including ZnO, Fe_3_O_4_, NiO, SnO_2_, ZrO_4_, TiO_2_, MgO and MnO_2_ which have been extensively explored for the detection of heavy metals due to their exciting nano‐morphological, functional biocompatible, non‐toxic and catalytic properties. These materials exhibit enhanced electron‐transfer kinetics and strong adsorption capability [15]. Among these nanomaterials, the ‘α’ polymorph of MnO_2_ nanostructures has been reported to possess superior catalytic performance owing to variable oxidation states of Mn centres [16, 17]. Furthermore, the non‐toxic and easy‐to‐synthesize nature of α‐MnO_2_ make them suitable towards the development of electrochemical sensing platforms [18, 19, 20]. The efficacy of α‐MnO_2_ towards heavy metal detection was evaluated by Zhang et al. who reported the second highest sensitivity achieved for Pb sensing (after Cadmium) [18]. Meanwhile, the performance of MnO_2_ nanostructures for heavy metal detection has further been enhanced by employing them as nanocomposites. For example, Wen et al. reported the use of nitrogen‐doped reduced graphene oxide and its composite with MnO_2_ nanostructures for Hg^2+^ detection with Limit of Detection (LoD) as low as 0.0414 nM [21]. In another work by Yang et al., α‐MnO_2_/Au nanocomposites were reported for trace analysis of As(III) [22]. However, to the best of our knowledge, the efficacy of α‐MnO_2_/GQD (GQD stands for Graphene Quantum Dots) nanocomposites has not been explored towards heavy metal detection and certainly not towards device applications for real‐time monitoring.

In the current study, we report ultrasensitive detection of Pb by employing α‐MnO_2_/GQD nanocomposites modified Au micro‐electrodes and packaging the electrode unit into a portable module with miniaturized electronics, capable of displaying the quantities of Pb(II) detected. It was envisaged that the superior catalytic performance of α‐MnO_2_ and high surface area of GQD would aid in enhanced complexation of Pb with the surface oxygen, followed by oxidation of Pb and high electron transfer kinetics through the sp^2^ backbone of GQD, thereby yielding an efficient and sensitive platform for real‐time monitoring of Pb(II) for on‐field applications. We have also validated the response, obtained from the developed portable resistance sensor, by employing Electrochemical Impedance Spectroscopy (EIS) in terms of LoD.

## 2 EXPERIMENTAL

### 2.1 Chemicals and reagents

Manganese chloride (MnCl_2_), potassium permanganate (KMnO_4_) and lead acetate (Pb(C_2_H_3_O_2_)_2_) were purchased from Sigma Aldrich Pvt Ltd, USA, while citric acid (C_6_H_8_O_7_) powder, sodium hydroxide (NaOH) and isopropyl alcohol (IPA) were obtained from Sisco Research Laboratories Pvt Ltd, India. All the chemicals were of analytical grade (purity ∼ 99.2%) and did not require further purification.

### 2.2 Synthesis of GQDs

The synthesis of GQDs was performed as per the protocol adopted by Dong et al. [23]. Briefly, 2 g powdered C_6_H_8_O_7_ was weighed and transferred in a 25 ml beaker followed by heating at 200°C. After 10 min, the powdered form transformed into the liquid phase, along with a colour change from colourless to pale yellow. In another 250 ml beaker, 100 ml of 10 mg/ml solution of NaOH was prepared. For aqueous solution of GQDs, the liquid phase of C_6_H_8_O_7_ was added drop wise into the NaOH solution with continuous stirring for 20–‐30 mins, until the pH was neutralized (pH ∼ 7).

### 2.3 Synthesis of α‐MnO_2_/GQD nanocomposite

The nanocomposite was synthesized by adding a fixed quantity of prepared GQD solution during the synthesis of α‐MnO_2_ nanofibres, the latter being adopted from a study by Kumar et al. [24]. An aqueous solution was made by adding 5.785 g of MnCl_2_ in 17 ml double distilled water (DDW) (Solution A). For Solution B, 3.475 g of KMnO_4_ was added in 56 ml DDW followed by 1.7 ml of 1 concentrated HNO_3_. Now, 5 ml of the prepared GQD solution was added to Solution A and placed in an ultrasonic chamber for 20 min. Solution B was then added to the modified Solution A drop wise with continuous stirring. Further, the resultant mixture was refluxed at 100 C for 16 h. The precipitate with black‐brown appearance was filtered and then washed multiple times with double distilled water till pH of the filtrate was neutralized. The final product (α‐MnO_2_/GQD) was dried for 12 h at 120 C followed by 6 h of calcination at 300 C.

### 2.4 Characterization of α‐MnO_2_/GQD nanocomposite

Scanning Electron Microscopy (SEM) images of α‐MnO_2_/GQD were acquired using Zeiss SEM model‐EVO18 at a beam energy of 20 keV interfaced with EDX (Energy Dispersive X‐ray analysis) module; the latter being employed for elemental analysis. Further clarity on the morphology of the nanocomposite was achieved from Transmission Electron Microscopy (TEM), which was performed at a beam energy of 120 keV. On the other hand, molecular fingerprint and functional group identification were analysed using Fourier Transform Infrared Spectroscopy (FTIR) within 4000 cm^−1^ to 500 cm^−1^. The spectrum was recorded by Perkin Elmer FTIR L125000P interferometer.

### 2.5 Sensor fabrication

**FIGURE 1:**
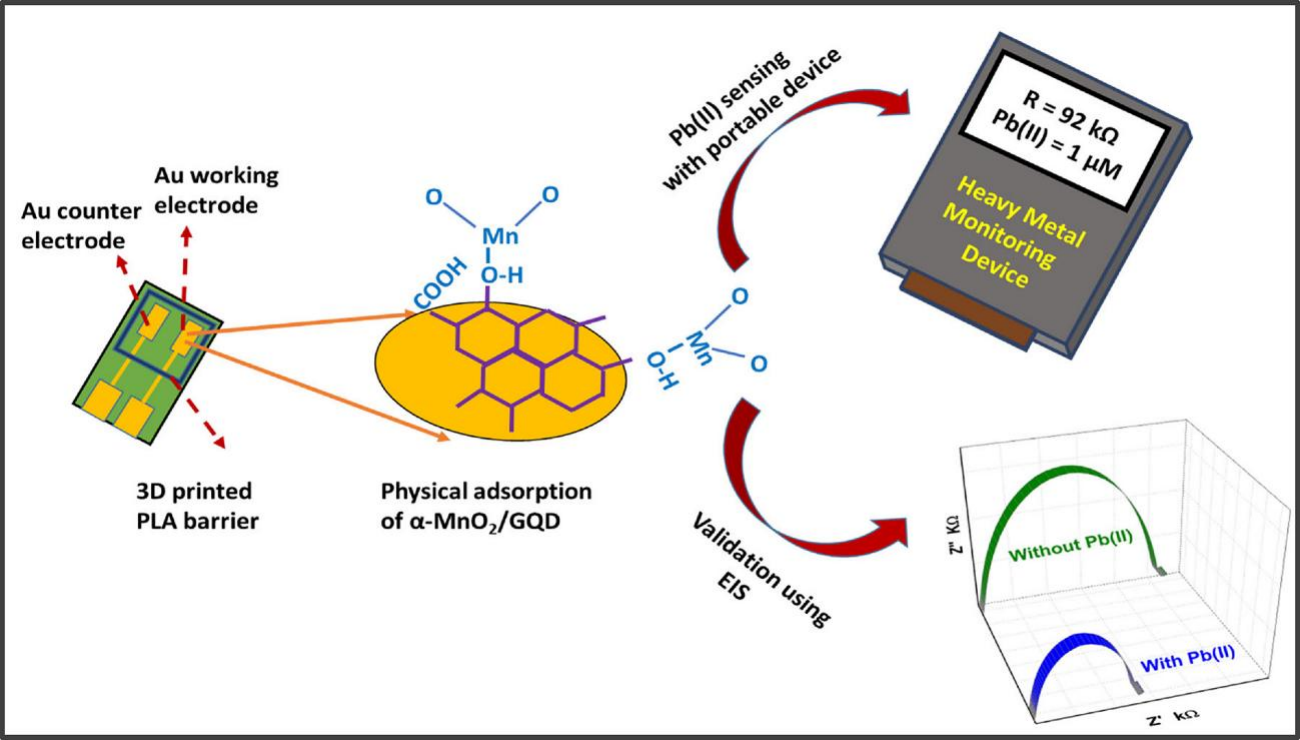
Schematic of nanosensor fabrication and the Pb(II) detection strategy using portable resistive device and validation with the impedance analyser (EIS)

The Au‐coated electrodes were fabricated using standard photolithography technique [25]. The working electrode, of area ∼0.5 cm^2^, was coated with 50 μL suspension of α‐MnO_2_/GQD (25 mg/ml in IPA) and left to dry for 2 h at room temperature. Finally, a 3D printed barrier composed of poly‐lactic acid (PLA) filament was sealed over the working and counter electrodes forming an electrolytic cell. The PLA barrier was allowed to settle for 24 h, at room temperature, which ensured complete sealing and enclosure of the working and counter electrodes as shown in Figure 1. Analyte volume of 200 μL each, consisting of 0.001 nM–1 μM Pb(II) in De‐Ionized (DI) water (pH ∼ 7), was precisely dropped in the cell in order to perform the electrochemical sensing analysis as displayed in Figure 1. It should be noted that separate electrodes were employed for electro‐analytical sensing studies, for each Pb(II) concentration, in order to avoid the memory effects due to adsorbed products (on a single sensor surface) as a result of Pb(II) oxidation.

## 3 RESULTS AND DISCUSSION

### 3.1 Electrode surface characterization

**FIGURE 2:**
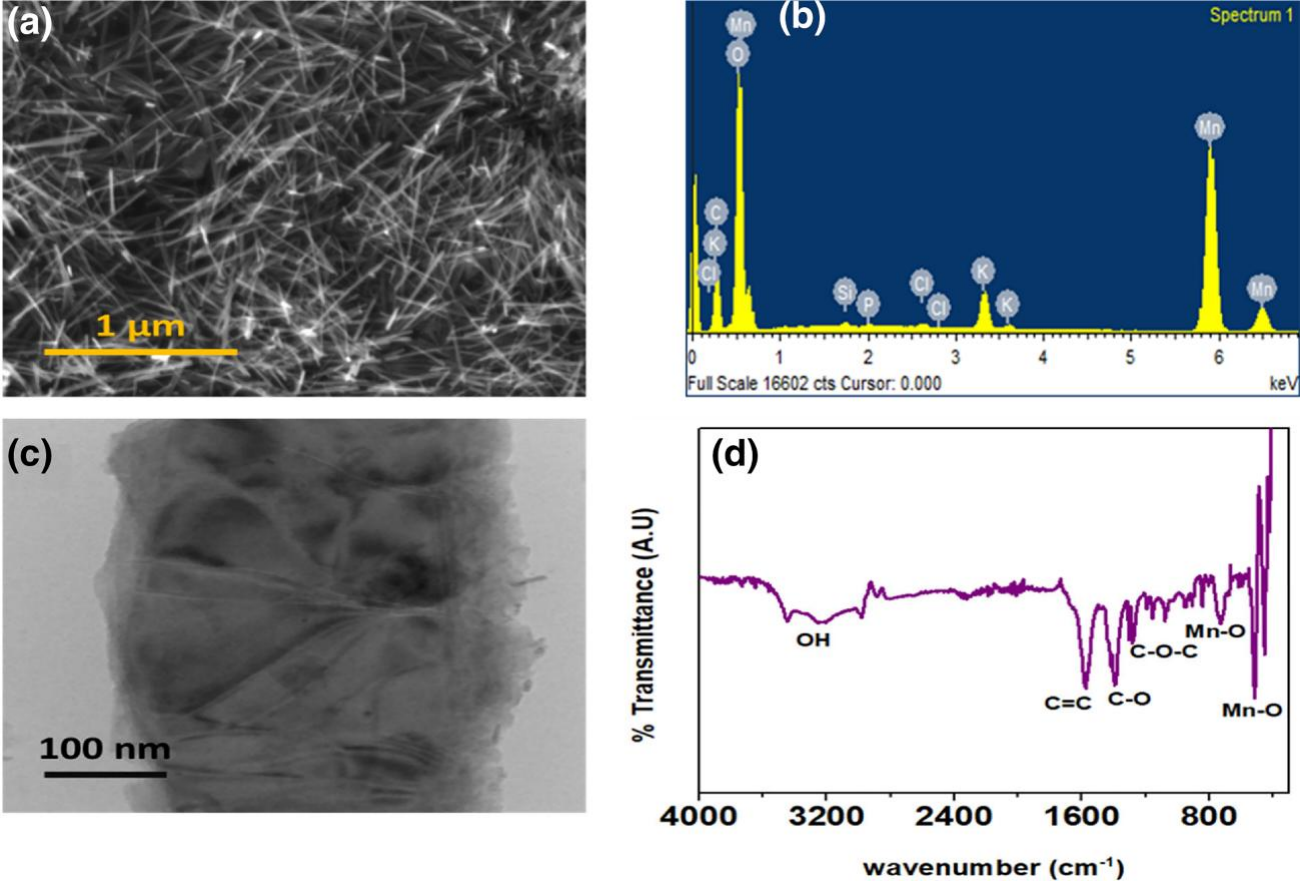
(a) SEM micrograph recorded at 20 keV, (b) EDX spectrum, (c) TEM image obtained at 120 keV, (d) FTIR spectrum obtained within 4000 cm^−1^ to 500 cm^−1^ of α‐MnO_2_/GQD nanocomposites

Figure 2a shows the SEM image of α‐MnO_2_/GQD nanostructures. The morphology reveals uniformly distributed nanoparticles with nanofibrous morphology. The average diameter and length of α‐MnO_2_ nanofibres were found to be ∼45 nm and ∼1 μm, respectively, which are consistent with those reported earlier [17]. On the other hand, the EDX spectrum in Figure 2b shows the presence of Mn and C as the dominant species in the composite, corresponding to α‐MnO_2_ and GQD, respectively.

The transmission electron micrograph (Figure 2c), imaged at a single nanofibre shows a layered structure of the MnO_2_‐GQD nanocomposite. It is possible to verify that the graphite structure is formed by several thick layers arranged in stacks. Moreover, the oxidation of graphite caused changes in its morphology, with a wrinkled appearance with several folds. It can be observed that this product consists of nanosphere/nanofibre hierarchical nanostructures with average nanodot diameter ∼10 nm. The FTIR spectrum of α‐MnO_2_/GQD nanostructures, as shown in Figure 2d, demonstrated a broad envelope between 3400 cm^−1^ and 3000 cm^−1^, corresponding to OH stretching vibrations. The doublet around 500 cm^−1^ and a singlet peak at 718 cm^−1^ can be attributed to the Mn‐O bending modes of octahedral MnO_6_ structures [26], while the presence of GQDs in the fibre matrix was confirmed by the C=C, C‐O and C‐O‐C bending vibrations in graphitic plane at 1580 cm^−1^, 1377 cm^−1^ and 1276 cm^−1^, respectively [27], thereby confirming the formation of MnO_2_/GQD nanocomposites.

### 3.2 Electroanalytical detection of Pb(II) in water

#### 3.2.1 Pb(II) sensing using portable device

**FIGURE 3:**
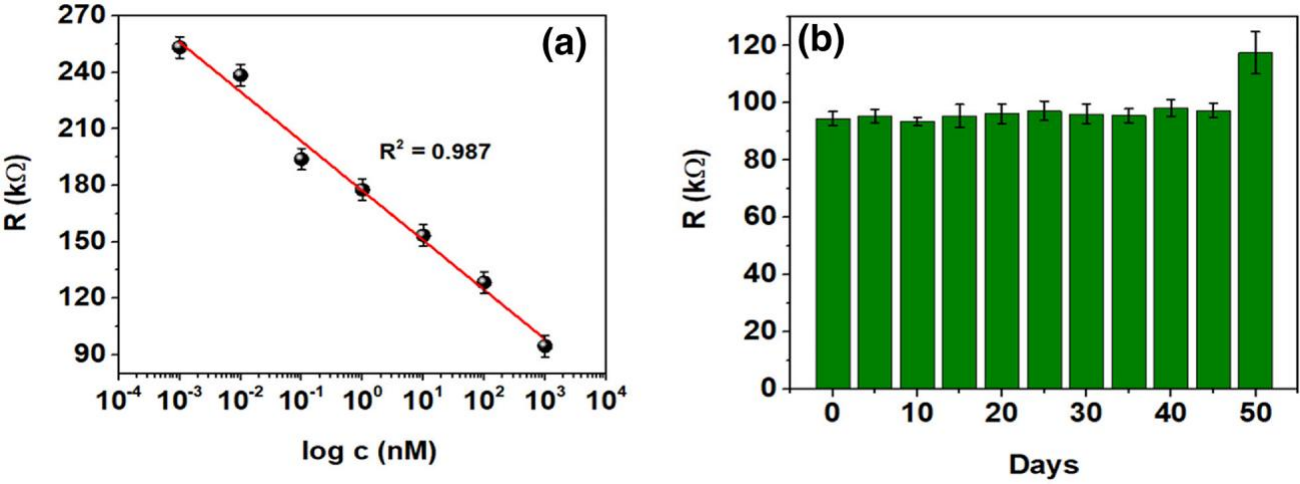
(a) Sensor calibration of Pb(II) detection (0.001 nM–1 μM) at 500 mV using portable resistive device, (b) Shelf‐life analysis of Pb(II) sensor performed over 50 days at regular intervals of 5 days 1 μM Pb(II) has been used for shelf‐life studies

The electrochemical detection of Pb(II), in DI water, was performed in a two‐electrode setup within 0.001 nM to 1 μM using the portable resistive device operating at a DC voltage of 5 V. The device basically incorporates a voltage divider circuit, in order to reduce the huge 5 V DC to ∼500 mV, the latter potential being applied at the α‐MnO_2_/GQD modified electrode (working electrode). The sensing strategy is highlighted in Figure 1. The calibration plot in Figure 3a indicates the establishment of inverse proportionality between logarithm of Pb(II) concentration (*c*) and the observed resistance arising due to electro‐oxidation of Pb(II), and the ensuing charge transfer at the interface. The regression line equation was found to be *R*(kΩ) = −26.317 log c(nM) + 177.107, *R* ^2^ = 0.987.

It can be observed that the sensor resistance decreases from ∼255 to ∼90 kΩ as Pb(II) concentration is increased from 0.001 nM to 1 μM. The detection mechanism of Pb(II) at α‐MnO_2_/GQD surface (S) is shown in reactions R1 and R2.

The first step (R1) involves the chemical adsorption and consequent complexation of metal ions (Pb^2+^ in our case) by surface‐adsorbed OH groups, as reported by Ren et al. and Khanna et al. [28, 29]. These OH groups arise due to trace amounts of adsorbed moisture on the surface, as evidenced by the broad envelope at around 3200 cm^−1^ in the FTIR spectrum (Figure 2d). The rapid reduction of Pb^4+^ to Pb^2+^ by Mn^2+^ has already been reported by Shi et al. [30]. Similarly, it is possible for Pb^2+^ to get rapidly oxidized by Mn^4+^ (on the surface S) within the cationic complex to form Pb^4+^. This type of adsorption mechanism was also reported by Scott and Morgan for Se(IV) oxidation to Se(VI) on the surface of δ‐MnO_2_ [31]. This is the second step as indicated by reaction R2 and involves the exchange of two electrons via an inner sphere pathway, which leads to rapid charge transport through the π‐conjugated backbone of GQDs. This process increases rapidly at higher Pb^2+^ concentrations due to a greater degree of Pb^2+^ adsorption onto the surface oxygen species, which act as Lewis acids by accepting more electrons [28] (leading to enhanced electron exchange between Mn and Pb), and ultimately decreasing the sensor resistance to 90 kΩ at 1 μM Pb(II). The LoD (using the conventional 3σ rule) and sensitivity of the portable Pb(II) sensing device was calculated to be 0.81 nM and 1.05 kΩ/nM/mm^2^, respectively [32].

Furthermore, the stability of the developed portable sensor, towards Pb(II) detection, was assessed by studying its shelf‐life for 50 days at intervals of 5 days as shown in Figure 3b. It can be seen that the value of *R*, at 1 μM Pb(II), obtained over 45 days is ∼95.47 ± 2.65 kΩ after which a significant increase in *R* was observed. This indicates the stability of the proposed portable sensor over 45 days, thereby confirming the reproducibility and shelf‐life within the specified period.

### 3.3 Validation of portable sensor response using EIS

**FIGURE 4:**
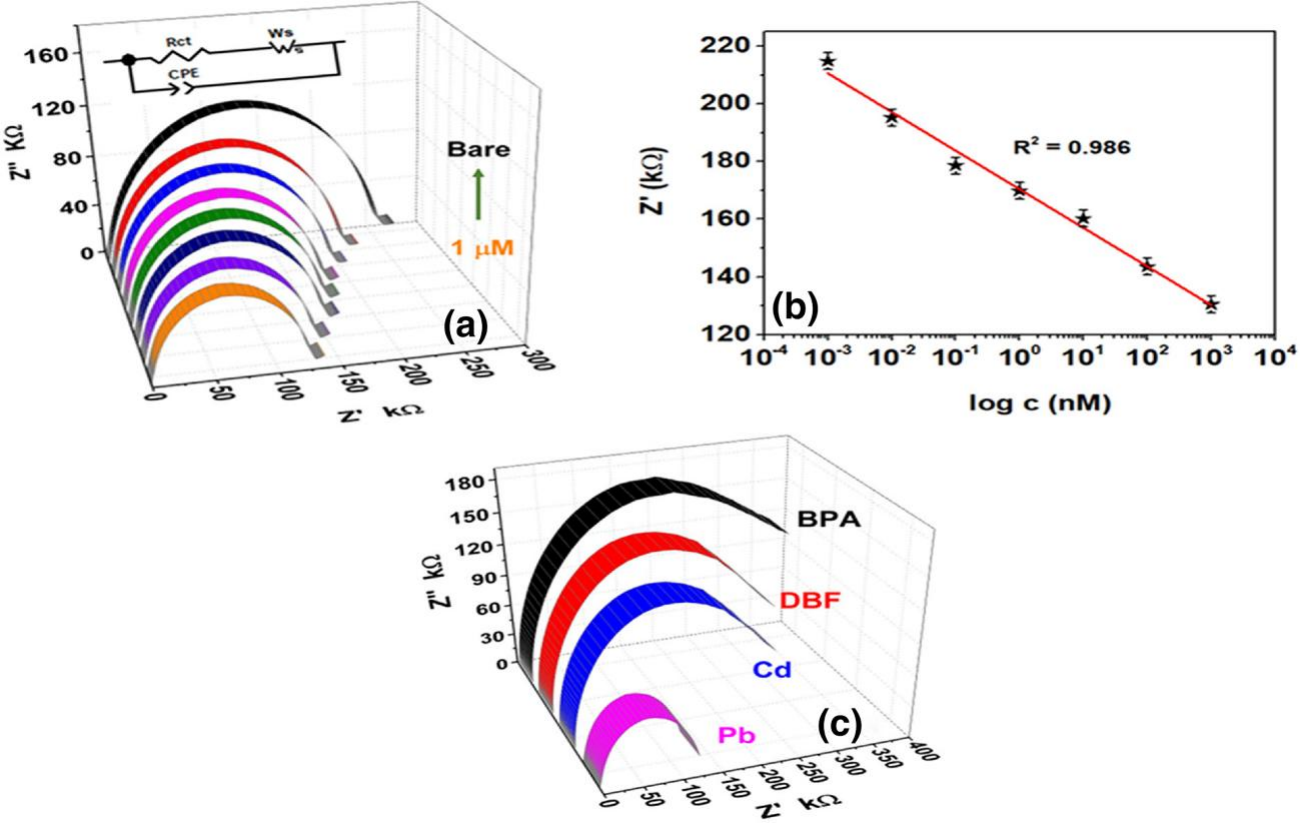
(a) Nyquist plot at various Pb(II) concentrations in the range 100 Hz–1 MHz at 100 mV AC, (b) Sensor calibration within 0.001 nM–1 μM Pb(II) at 20 kHz, (c) Nyquist spectra of Pb, Cd, DBF (Dibenzofuran) and BPA (Bisphenol‐A) highlighting the effects of potential interferants towards Pb detection. The concentration of each of the chosen compounds for interference analysis is 1 µM

In order to validate the developed portable Pb(II) sensor, the α‐MnO_2_/GQD modified electrodes were employed into a two‐electrode setup for performing impedance spectroscopy. The frequency was swept between 100 Hz and 1 MHz at a fixed sinusoidal bias of 100 mV, while DI water was incorporated as the electrolyte. The impedance response (Figure 4a,b) obtained from EIS indicates a decrease in sensor impedance as the concentration of Pb(II) is increased to 1 μM. The decrease in the Alternating Current (AC) impedance is consistent with the DC resistance observed at higher Pb(II) concentrations (Figure 3a).

The Nyquist plot in Figure 4a indicates a semi‐circular feature and a straight line 45° to *Z′* axis. The semi‐circle on left‐hand side corresponds to the charge transfer at α‐MnO_2_/GQD surface from DI water electrolyte, through an imperfect electrical double layer capacitor [33, 34]. This is modelled by the elements *R*_ct_ and CPE respectively in Randel’s circuit depicted in Figure 4a inset. The straight line describes low frequency diffusion kinetics of electrons through tunnels of α‐MnO_2_ which can be modelled by Warburg impedance *W*_s_ (Figure 4a) [35]. The diameter of semi‐circle, *R*_ct_ (*Z*′), can be observed to decrease at higher Pb(II) levels. As shown in reactions **R1** and **R2**, the latter enriches the electrode‐electrolyte interface with protons (H^+^) and electrons respectively at higher concentrations which enables rapid charge transport (low R_ct_) at α‐MnO_2_/GQD surface from electrolyte, and therefore justifies the low resistance response (*R*) observed at elevated Pb (II) levels (as shown in Figure 3a). The height of the half‐semicircle gives a measure of the double layer capacitive impedance Z’’. The effect of low *R*_ct_ from EIS (low *R* from portable device) at a higher Pb concentration indicates the increase in the number of electrons and H^+^ ions at the interface. In other words, the latter gets filled with charges, leading to enhanced double layer charging, which results in an increase in double layer capacitance and a consequent decrease in *Z*’’ [36]. Furthermore, the decrease in diffusion impedance at high Pb(II) levels indicates rapid interfacial electron transfer (low *R*_ct_) through the tunnels of α‐MnO_2_, thereby validating the sensor response obtained using the portable device. The sensor was calibrated at 20 kHz, by analysing *Z*’ (*R*_ct_) as a function of Pb(II) concentration (*c*), while the latter was varied from 0.001 nM–1 µM. The calibration graph (Figure 4b) indicates that *Z*′ was found to be inversely proportional to Pb(II) concentration within the specified concentration range of the analyte, with regression line equation of *Z*′(kΩ) = −13.38log *c* (nM) + 170.40 and *R* ^2^ = 0.986 along with a sensitivity of 0.53 kΩ/nM/mm^2^. Apart from the consistency of the linear trend observed in the sensor calibration of the EIS and the portable device, it is worthwhile to note that the LoD from the former technique was calculated to be 0.84 nM which is almost equal to that obtained from the resistance sensing device. This observation further led us to conclude that the developed portable Pb(II) monitoring device, based on α‐MnO_2_/GQD nanocomposites, is highly suitable for real‐time and on‐field applications. Table 1 shows the comparison of LoDs and sensitivities obtained from EIS and the developed Pb(II) platform.

**TABLE 1:** Comparison of LoD and sensitivity calculated using the EIS and the developed portable Pb(II) monitoring device

Sl. No	Pb(II) Sensing Strategy Employed	LoD (nM)	Sensitivity (kΩ/nM/mm^2^)
1	Portable resistive device	0.81	1.05
2	Commercial EIS	0.84	0.53

Furthermore, the effect of potential interferants found in water bodies namely, Cadmium (Cd), Dibenzofuran (DBF) and Bisphenol‐A (BPA) (1 µM each) was also studied on the response of Pb(II) detection as shown in Figure 4c. The Nyquist spectra indicates distinct impedance response for each analyte with significant differences observed between Pb and the chosen potential interferants, due to reactions **R1** and **R2** being native solely to Pb(II), thereby validating the efficacy of the latter to be employed in real‐time monitoring of Pb(II) in water bodies.

## 4 CONCLUSION

An electrochemical, label‐free portable resistive sensor for the detection of Pb(II) in water, employing α‐MnO_2_/GQD as receptor was developed. The portable sensor displayed prominent linear characteristics at 500 mV DC, within a wide analyte concentration range of 0.001 nM–1 µM, thereby establishing a direct proportionality between the sensor resistance and Pb(II) concentration. The limit of detection (LoD) and sensitivity of the developed sensor was calculated to be 0.81 nM and 1.05 kΩ/nM/mm^2^, respectively, which was verified by the commercial impedance analyser; while the latter exhibited a linear nature within 0.001 nM–1 μM Pb(II) with LoD and sensitivity of 0.84 nM (approximately equal to that obtained with portable device) and 0.53 kΩ/nM/mm^2^, respectively. The presence of potential interferants, found in water bodies, was found to have insignificant impact on the Pb(II) response. The developed platform was further found to generate a stable resistance response up to 45 days, thereby confirming the sensor stability for more than a month. Table 2 compares the developed Pb(II) sensor with those reported in the literature. The ultra‐low LoD along with superior stability of the developed sensor indicated its potential application in real‐time and on‐field monitoring of Pb(II).

**TABLE 2:** A comparison of the developed Pb(II) sensors with those reported in the literature

Electrode	Detection Technique	LOD (μg L^−1^)	Reference
Bi/poly(1,8‐diaminonaphthalene) modified carbon paste electrode	SWV	0.3	[9]
Bi nanoparticles/Nafion®‐modified pencil graphite electrodes	ASV	31.1	[37]
Screen‐printed carbon electrodes	SWASV	2.0	[38]
Cu electrode	ASV	4.4	[39]
Bi–C nanocomposite	SWASV	0.7	[40]
BiNPs	SWASV	2.0	[41]
CoFe_2_O_4_/Bi nanocomposite	SWASV	1.5	[42]
SWCNT/screen printed electrodes	SWASV	0.4	[43]
α‐MnO_2_/GQD/Au electrodes	EIS	0.3 (0.81 nM)	Present work

*ASV = Anodic stripping voltammetry; SWV = Square wave voltammetry; SWASV = Square wave anodic stripping voltammetry.

## CONFLICTS OF INTEREST

The authors declare no conflict of interests.
